# Outburst of pest populations in rice-based cropping systems under conservation agricultural practices in the middle Indo-Gangetic Plains of South Asia

**DOI:** 10.1038/s41598-022-07760-w

**Published:** 2022-03-08

**Authors:** Rakesh Kumar, Jaipal Singh Choudhary, Janki Sharan Mishra, Surajit Mondal, Shishpal Poonia, Mohammad Monobrullah, Hansraj Hans, Mausam Verma, Ujjwal Kumar, Bhagwati Prasad Bhatt, Ram Kanwar Malik, Virender Kumar, Andrew McDonald

**Affiliations:** 1 ICAR-Research Complex for Eastern Region, Patna, Bihar India; 2 ICAR-Research Complex for Eastern Region, FSRCHPR, Ranchi, Jharkhand India; 3 ICAR-Directorate of Weed Research, Jabalpur, Madhya Pradesh India; 4 Cereal Systems Initiative for South Asia (CSISA)-CIMMYT, Patna, India; 5 Indian Council of Agricultural Research, New Delhi, India; 6 International Rice Research Institute, Los Banos, Philippines; 7 Soil and Crop Sciences Section, School of Integrative Plant Science, Cornell University, Ithaca, NY USA

**Keywords:** Climate sciences, Environmental sciences

## Abstract

Conservation agriculture (CA), which encompasses minimum soil disturbance, residue retention either through crop residue, or cover crops and crop diversification-based crop management practices can modify the status of pest dynamics and activities under the changing climatic scenarios. CA has been advocated extensively to optimize the use of available resources, maintain the environmental quality, enhance crop productivity, and reduce the climate change impacts. Information related to the impacts of long-term CA-production systems under rice-based cropping systems on pest status is lacking, particularly in middle Indo-Gangetic Plains (MIGP). Under CA, puddling is completely avoided, and rice is directly sown or transplanted to maintain better soil health. Different sets of experimentations including farmers practice, partial CA and full CA (CA) as treatments in rice-based cropping systems, were established from 2009, 2015 and 2016 to understand the long-term impacts of CA on pest dynamics. In this study, direct and indirect effects of tillage (zero, reduced and conventional tillage), residue retention and cropping sequences on abundance and damage by pests were investigated. After 4–5 years of experimentation, populations of oriental armyworm [*Mythinma (Leucania) (Pseudaletia) separata* (Wlk.)] in wheat*,* mealybug [*Brevennia rehi* (Lindinger)] and bandicoot rat [*Bandicota bengalensis* (Gray)] in rice were found to increase abnormally in CA-based production systems. Conventionally tilled plots had a significant negative effect while residue load in zero-tilled plots had a significant positive effect on larval population build-up of *M. separata*. Zero tillage had a higher infestation of mealybug (52–91% infested hills) that used grassy weeds (*Echinochloa colona*, *Echinochloa crusgalli*, *Cynodon dactylon*, *Leptochloa chinensis* and *Panicum repense*) as alternate hosts. Cropping sequences and no disturbance of soil and grassy weeds had higher live burrow counts (4.2 and 13.7 burrows as compared to 1.47 and 7.53 burrows per 62.5 m^2^ during 2019–2020 and 2020–2021, respectively) and damaged tillers (3.4%) in CA-based practices. Based on the present study, pest management strategies in CA need to be revisited with respect to tillage, residue retention on soil surface, grassy weeds in field and cropping sequences to deliver the full benefits of CA in MIGP to achieve the sustainable development goals under the climate change scenarios.

## Introduction

The Middle Indo-Gangetic Plains (MIGP) covering eastern Uttar Pradesh and Bihar are endowed with rich and diverse natural resources but has lower crop productivity and per capita income^1^. Rice-based cropping systems are the major production systems of the region occupying an area of 10.5 M ha^2,3^. Due to small-fragmented landholdings and resource-poor farmers, agricultural production systems of the MIGP are less mechanized^4,5^ and highly labour intensive as compared to the north-western part of the IGP^6–8^. Major crops grown in the MIGP in rotation with rice are wheat, maize, oilseeds, and pulses. Conventional crop production practices rely upon the high inputs in terms of machinery, labour, nutrients, water, and chemical pesticides^9–11^, which also leads to soil degradation through erosion, compaction, decreased water-holding capacity, and loss of soil organic carbon (SOC). These practices also alter the habitats of surface and sub-surface micro- and macro-biota and their niches^12,13^. Thus, there is a pressing need to develop a sustainable, economically viable and eco-friendly intensified and diversified cropping system, and crop management options for the MIGP.

As a technological innovation, CA-based production systems, which retains residue of previous crops, minimum mechanical disturbance of the soil, and crop diversification, are being promoted in the MIGP^14,15^. CA practices not only enhance the soil quality but also favour the habitats for soil-dwelling organisms^16,17^. Overall, CA is an approach to crop production for enhancing and sustaining the production, increasing profits and achieving the food security^18,19^. However, many challenges of CA-based production systems still need to be identified and addressed within the regional context to realize the maximum benefits^20–23^. Applying CA-based management principles within crop production not only changes soil organic matter (SOM), soil moisture, and nutrient regimes but also shifts the diversity of certain pests^24–27^. Pests are the major threat to crop production and their status changes with the adopted management practices in agro-ecosystem^13,28^. Since applying CA-based practices within an agroecosystem might shift pest density and diversity^13^, thus, the pest species that can adjust to certain CA management practices will outburst, while others will disappear.

Tillage management practices and residue retained on the soil surface alter the microclimate within the crop canopy of CA-based systems; therefore, certain insects will grow and proliferate, while others may disappear or decrease depending upon the pest concerned. Long-term solutions to pests can be provided through effective integration of tillage management with other alternative methods such as cultural, mechanical, physical, and biological. For example, integration of CA practices in the field can be effectively utilized for the augmentation of natural enemies of insect pests. Similarly, covering the soil with residue may accelerate the population density of many insect pests and other arthropods, but an increased population of generalist predators may be suppressing^29,30^. Therefore, shifting from conventional production practices to CA practices may change insect-pest scenarios and management strategies due to changes in micro-ecology^31^.

Alterations in pest abundance and subsequent crop damage are the most challenging issues of CA^13^. Soil and crops are important habitats for pests and diseases, and we hypothesized that change in tillage and residue retention patterns in CA in comparison to conventional practices may have some noticeable positive and negative impacts on the pest dynamics. A report of rice mealybug, *B. rehi,* from a long-term rice-based CA production system in MIGP indicates the potential threat of insect pests and disease in CA; thus, any changes in pest dynamics need to be reported in scientific literature to advance the management strategies^26^. Therefore, the present study was undertaken to have a better understanding of potential emerging issues of pests in long-term CA-based systems in MIGP and to explore their possible relationship with tillage and residue management in cropping sequences.

## Materials and methods

### Site description and weather conditions

**Figure 1:**
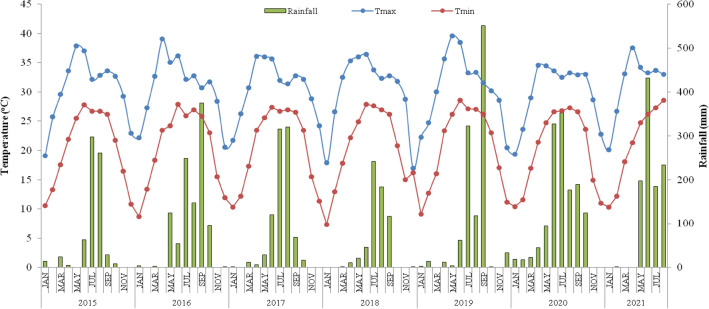
Monthly total rainfall and mean monthly maximum and minimum temperatures prevailed during the experimental period (January 2015 to July 2021).

Long-term rice-based CA experiments were established from 2009, 2015 and 2016 at the Research Farm of Indian Council of Agricultural Research (ICAR)— Research Complex for Eastern Region (RCER) (25° 35′ N, 85° 05′ E, and 51 m above mean sea level) Patna, Bihar, India. Soil (Vertic Endoaqualfs) is silty loam in texture (22% sand, 54% silt and 24% clay); pH 7.22; organic carbon 6 g kg^−1^; electrical conductivity 0.17 dS m^−1^; available N 188 kg ha^−1^; available P 12.9 kg ha^−1^; and available K 137 kg ha^−1^. Weather parameters were recorded during the study period (2015–2021) and are presented in Fig. 1. The climate of the experimental site is sub-tropical humid with an average annual rainfall of 1127 mm (85–90% of which was received during June to September). The distribution of rainfall over time and intensity in rainy season was very erratic. The lowest rainfall (621 mm) was recorded in 2018, and the highest rainfall (1367 mm) in 2020. The lowest minimum temperature (7.4 °C) was recorded in January 2017, and the highest (41.3 °C) in May 2018. Generally, the maximum temperature exceeding 35 °C was noted in April, May, and June and the lowest in January. The rise and fall of maximum temperature were controlled by thunderstorm activity in summer, and that of the minimum temperature was controlled by the passage of western disturbance in winter.

### Treatment details and experimental design

Broadly treatments were covered under three practices, viz*.* conventional tillage as farmers practice (FP), partial CA (pCA) and full CA (CA). The first set of CA experiments was established in 2009, where conventional tilled (CT) puddle transplanted rice (TPR)-wheat (CT)–fallow (FP), CT-mechanical transplanted rice (MTR)–wheat (ZT)–mungbean (ZT) (pCA), zero-till direct seeded rice (ZTDSR)–wheat (ZT)–mungbean (ZT) (CA) and ZTDSR-mustard (ZT)–ZT spring maize (CA) were followed^1,18^ (Supplementary Table 1). The second set of experiments based on diversified CA cropping systems was established in 2015, where an experiment comprising seven tillage and crop establishment (TCE) methods for a rice–wheat-mungbean system was established in a randomized complete block design (RCBD) with three replications. Briefly, seven treatments in different management practices were divided as follows: Sc1) random puddled transplanted rice (RPTR)-conventional tilled (CT) broadcast wheat (BCW)-zero-till mungbean (ZTM); Sc2) line puddled transplanted rice (LPTR)-CT drilled wheat (CTW)-ZTM; Sc3) conventional tilled machine transplanted rice (CTMTR)-zero-till wheat (ZTW)-ZTM; Sc4) zero-till machine transplanted rice (ZTMTR)-ZTW-ZTM; Sc5) system of rice intensification (SRI)-system of wheat intensification (SWI)-ZTM; Sc6) CT direct-seeded rice (CTDSR)-ZTW-ZTM; Sc7) zero-till DSR (ZTDSR)-ZTW-ZTM^15,20^ (Supplementary Table 2). The third set of CA experiments was established in the rainy season of 2016 to address the rice-fallow production system, where rice-chickpea, rice-lentil, rice-safflower, rice-linseed, and rice-mustard sequences were followed under diverse tillage production systems (ZTDSR, CTDSR and TPR with and without residue management practice^27^ (Supplementary Table 3). Agronomic management practices were followed in all the experiments, as rice was directly sown in rows 22.5 cm apart during the 3rd week of June every year by ZT Happy Seeder with 25 kg seed ha^–1^ at a 3–4 cm seeding depth in all DSRs (CT/ZT). Nurseries for PTR, MTR and SRI were raised on same day with the recommended package of practice. A mat-type nursery was raised for MTR with 20 kg seed ha^−1^ ^32^. For PTR and SRI, nursery beds were prepared with seed rates of 15 and 7 kg ha^–1^, respectively. Wheat (HD 2967) was sown during second fortnight of November. In all CT and ZT, wheat was sown in rows at 22.5 cm apart with 100 kg seed ha^–1^ using ZT Happy Seeder, except in CT-broadcast wheat (BCW) where the manual broadcasting and mixing with rotavator was done with 120 kg ha^–1^, and SWI where seeds were manually dibbled with 25 kg seed ha^–1^. In summer, short duration (60–65 days) mungbean (Samrat) was sown under ZT condition immediately after wheat harvest using ZT Happy Seeder at 22.5 cm × 5 cm spacing with 30 kg seed ha^–1^ during the second week of April. Recommended doses of 120 kg N, 60 kg P_2_O_5,_ and 60 kg K_2_O ha^–1^ as urea, di-ammonium phosphate (DAP) and muriate of potash (MOP), respectively, were applied to rice and wheat. One-third of the recommended N and full doses of P and K were applied as basal. The remaining 2/3rd N was applied in two equal splits at the maximum tillering and panicle initiation stages. For mungbean, 100 kg DAP ha^–1^ was applied as a basal application through Happy Seeder. Pendimethalin (30% EC) at 1.0 kg *a.i.* ha^–1^ in DSR and pretilachlor at 0.75 kg *a.i.* ha^–1^ in TPR were applied as pre-emergence (2 DAS/DAT) while bispyribac-sodium at 25 g *a.i.* ha^–1^ was applied as post-emergence at 20 DAS/DAT. In wheat, a ready-mix combination of sulfosulfuron (75% WG) + metsulfuron methyl (5% WG) @ 32 (30 + 2) g *a.i.* ha^–1^ was applied as post-emergence (25 DAS). For weed control in mungbean, pendimethalin at 1.0 kg *a.i.* ha^–1^ was applied as pre-emergence (next day after seeding). A knapsack sprayer fitted with a flat-fan nozzle with 500 L ha^–1^ of water was used for applying the herbicides. Rice was irrigated depending upon the occurrence of dry spells during cropping. In wheat, irrigation was applied at crown root initiation (CRI), tillering, flowering, and grain filling stages. In mungbean, in addition to pre-sowing irrigation, two irrigation treatments at 25 and 45 DAS were applied. The authors confirm that experiments on plant species in different CA systems in the present study comply with the institute guidelines and legislations.

### Pest sampling

After the establishment of experiments, data on abundance and damage by major arthropod pests were taken to address the potential challenges of conservation agriculture (CA) in MIGP. Populations of oriental armyworm, *Mythinma (Leucania) (Pseudaletia) separata* (Wlk.) (Lepidoptera: Noctuidae) in wheat, rice mealybug, *Brevennia rehi* (Lindinger) (Hemiptera: Pseudococcidae) and bandicoot rat, *Bandicota bengalensis* Gray (Rodentia: Muridae) in rice were recorded after 3–4 years of the experiment establishment. The population densities of *M. separata* larvae and pupae were recorded in wheat at 65 DAS and after crop harvesting, respectively, placing a quadrate (1 m × 1 m) randomly at 5 places in each plot. Entire anchored residue in each quadrate were pulled out and larvae/pupae hiding inside crop residues were counted manually and averaged as number m^–2^. Infestation of rice mealybug in rice was recorded by counting the number of mealybug-infested hills in a running metre of a row in each treatment at the panicle formation stage. Infestation of mealybug was converted into percent infested hills. To establish the relationships between mealybug and weeds, total grassy weeds were counted in each year with the help of a quadrate (1 m × 1 m) placed randomly at five places in each treatment. Damage by bandicoot rats, *B. bengalensis* was assessed indirectly based on the presence of live burrows and directly by damaged tillers. The total number of live burrows and damaged tillers were counted visually for 62.5 m^2^ area in each treatment. Damaged tillers were converted into percent damage based on the total number of tillers.

### Statistical analysis

Data from the mealybug (*B. rehi)* and armyworm (*M. separata)* were analyzed using analysis of variance (ANOVA) according to Gomez and Gomez^33^ for randomized block design using SPSS software (SPSS 21). For homogeneity, data of mealybug and armyworm were arcsine and square root transformed, respectively, before statistical analysis. Treatment means were separated using *Tukey’s* honestly significant difference (HSD) at the 5% level of significance. Data on rodent burrow counts were analyzed by split-plot ANOVA at *p* = 0.05, where crop establishment-cum-residue management (CERM) was included as first factor and winter crop in sequences was included as the second factor. Mean effects of tillage and residue were determined using the linear contrast in SPSS 21. To determine any effect of weather parameters on pest outbreak, correlation analysis of the pest population under different CERM as dependent factor and weather parameters (minimum and maximum temperature and rainfall) as independent variables was done. All the figures were generated using XLSTAT^34^.

## Results

**Figure 2:**
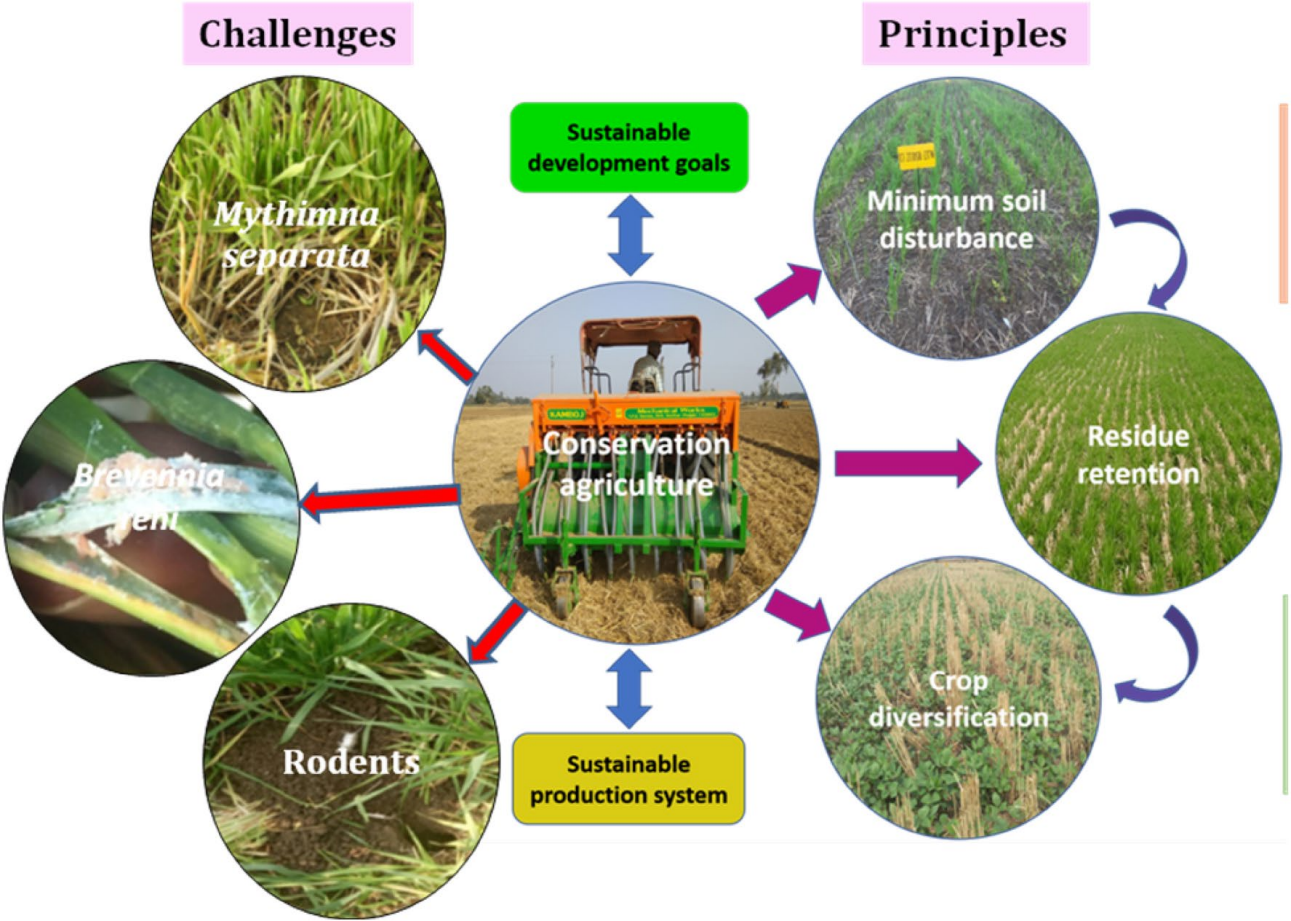
Schematic diagram of conservation agriculture (CA) production system with positivity and emerging issues in rice-based cropping systems of the middle Indo-Gangetic Plain.

Three arthropod species were observed as potential threats in long-term CA production systems after the establishment of experiments (Fig. 2). They were two insect species (Armyworm, *M. separata* & rice mealybug, *B. rehi*) and one rodent species (bandicoot rat, *B. bengalensis*).

### Armyworm, *M. separata*

**Figure 3:**
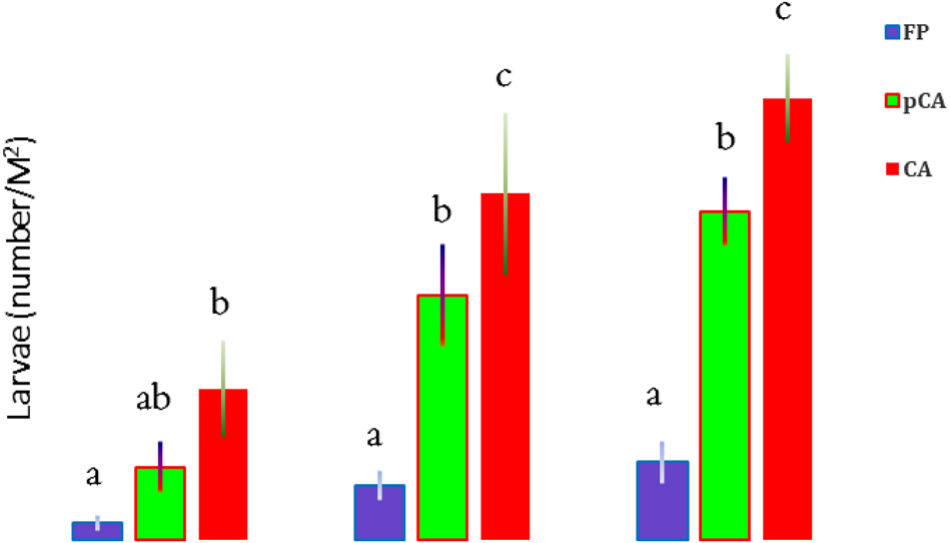
Mean infestation of *Mythimna separata* larvae on wheat in different tillage-cum crop establishment and residue management practices at 65 DAS during recent different years. Bars with different letters indicate significant differences among agricultural production systems (LSD; *P* < 0.05).

**Figure 4:**
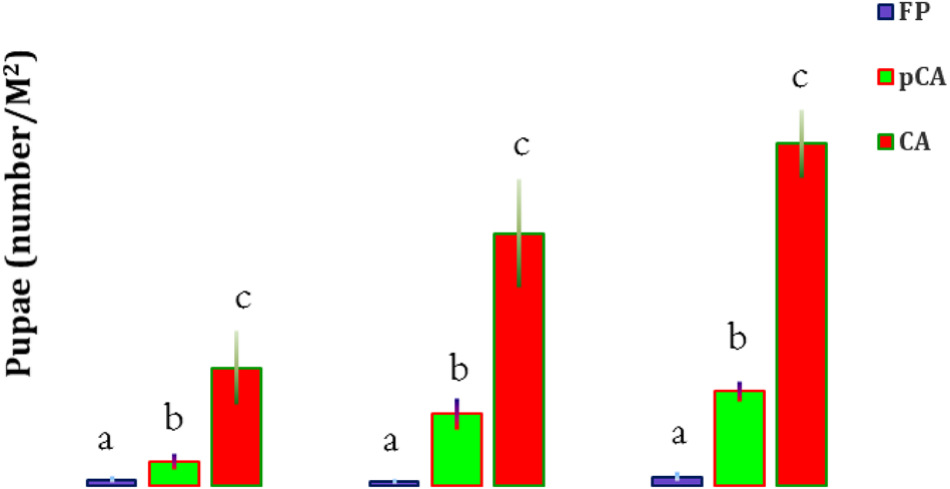
Mean number of pupae of *Mythimna separata* in the fields with different tillage-cum-crop establishment and residue management production systems after harvesting wheat in recent years. Bars with different letters indicate significant differences among production systems (LSD; *P* < 0.05).

Significant variations in larvae and pupae populations of *M. separata* were observed in wheat under different tillage-cum-crop establishment and residue management systems of CA (Figs. 3, 4). In CA-based production systems, a significantly (*p* < 0.05) higher population of armyworms in wheat was observed after 5th year of experimentation compared to conventional farmers practice (FP). The incidence of armyworm was first noticed in 2018–2019, after which the population increased over the years. The highest number of larvae was observed in CA-based production system (58.2 larvae/m^2^), followed by pCA (43.2 larvae/m^2^), and the lowest number (below ETL) was observed in FP (10.2 larvae/m^2^) in 2020–2021 in the first set of experiments (Fig. 3). In the second set of experiments (rice–wheat-mungbean), established in 2015, number of larvae and pupae of armyworm increased drastically during 2020–2021 as compared to 2018–2019 (Table 1). The maximum mean numbers of larvae (3.40 ± 0.93 m^−2^) and pupae (1.20 ± 0.49 m^−2^) were observed in treatment Sc7 during 2018–2019. In the same treatment (Sc7), the mean populations of larvae and pupae reached 10.4 ± 1.53 m^−2^ and 2.4 ± 0.24 m^−2^, respectively, during 2020–2021. The next maximum population of larvae and pupae was observed in another CA-based treatment Sc4 during the observation year, although it was not significantly different from treatment Sc7 (Table 1). The pCA based production systems (Sc3, Sc5 and Sc6) was recorded a mean population of larvae ranging from 6.8 to 7.6 m^2^ and was at par among them. The significantly lowest populations of larvae and pupae were observed in FP-based scenarios (Sc1 and Sc2) (Table 1).

**Table 1:** Infestation of *Mythimna separata* (mean numbers of larvae & pupae) on wheat crop and their correlations with incorporated/retained residue in different tillage-cum-crop establishment and residues management practices of Indo-Gangetic Plains at 65 days after sowing (larvae) and after harvesting of crop (pupae) during recent different years.

Scenarios*	Number of larvae (m^−2^)			Number of pupae (m^−2^)		
	2018–2019	2019–2020	2020–2021	2018–2019	2019–2020	2020–2021
Sc1 (FP)	0.20 ± 0.02^a^	0.80 ± 0.58^a^	1.60 ± 0.81^a^	0.00 ± 0.00^a^	0.20 ± 0.20^a^	0.40 ± 0.24^a^
Sc2 (FP)	0.60 ± 0.40^a^	0.60 ± 0.60^a^	2.00 ± 0.95^a^	0.20 ± 0.20^a^	0.40 ± 0.24^a^	0.60 ± 0.24^a^
Sc3 (pCA)	1.40 ± 0.51^ab^	4.20 ± 1.24^ab^	7.60 ± 1.43^b^	0.40 ± 0.24^a^	1.00 ± 0.32^ab^	1.60 ± 0.40^ab^
Sc4 (pCA)	3.20 ± 1.16^b^	6.60 ± 1.50^b^	9.60 ± 1.88^b^	1.00 ± 0.45^a^	1.60 ± 0.24^b^	2.00 ± 0.45^b^
Sc5 (pCA)	1.60 ± 0.81^ab^	5.00 ± 1.38^b^	6.80 ± 1.88^b^	0.40 ± 0.24^a^	1.00 ± 0.32^ab^	1.40 ± 0.51^ab^
Sc6 (CA)	2.20 ± 0.66^ab^	4.00 ± 1.30^ab^	7.40 ± 1.08^b^	0.40 ± 0.24^a^	0.80 ± 0.37^ab^	1.60 ± 0.24^ab^
Sc7 (CA)	3.40 ± 0.93^b^	8.00 ± 1.48^b^	10.40 ± 1.53^b^	1.20 ± 0.49^a^	1.80 ± 0.37^b^	2.40 ± 0.24^b^
Number of larvae/pupae (m^2^) regressed on residue incorporated/retained (r)						
Larvae (m^2^)				Pupae (m^2^)		
Y = 0.23x + 2.66 (R^2^ = 0.82*)				Y = 0.06X + 0.59 (R^2^ = 0.89**)		

Mean values followed by standard error and different superscript small letters within a column are significantly different; * and ** indicate significant at *P*<0.05 and <0.001, respectively.

Temperature (maximum and minimum) had played a significant role in population dynamics of *M. separata* (larva & pupae) but it was having non-significant effect among the different CERM practices (Supplementary Table 4). However, contrast analysis of tillage (till vs. no-till) and residue (residue retention vs. non-residue) on larval and pupal populations revealed differential effects (Table 2). CA-based practices had 9.0-, 6.3- and 5.7-times higher armyworm larval populations during 2018–2019, 2019–2020 and 2020–2021, respectively, than FP (Fig. 3). Tillage had a negative effect on the larval population during 2018–2019 and 2020–2021 (*p* = 0.02), while residue had a significant positive impact on the larval population build up during 2018–2020 (Table 2). Residue retention in CA-based systems also had a significant positive impact (*p* < 0.05) on pupae, as observed after wheat harvest. Residues retained in CA had 19.8, 56.3 and 38.2 times higher pupal populations during 2018–2019, 2019–2020 and 2020–2021, respectively, than FP (Fig. 4).

**Table 2:** Contrast analysis between years, crop establishment-cum-residue management and insect pest infestation.

Scenarios	*Mythimna separata*						Rice mealybug, *Brevennia rehi*		
	Larva			Pupa					
	2018–2019	2019–2020	2020–2021	2018–2019	2019–2020	2020–2021	2015	2016	2017
Till versus No-till	− **0.02**	− 0.08	− **0.02**	− 0.17	− 0.07	− 0.19	− 0.17	− **0.02**	− 0.07
Residue versus nonresidue	**0.01**	**0.04**	0.08	**0.002**	**0.01**	**0.05**	0.42	0.06	0.11

Values below 0.05 are significant and indicated in bold.

### Rice mealybug, *Brevennia rehi*

**Figure 5:**
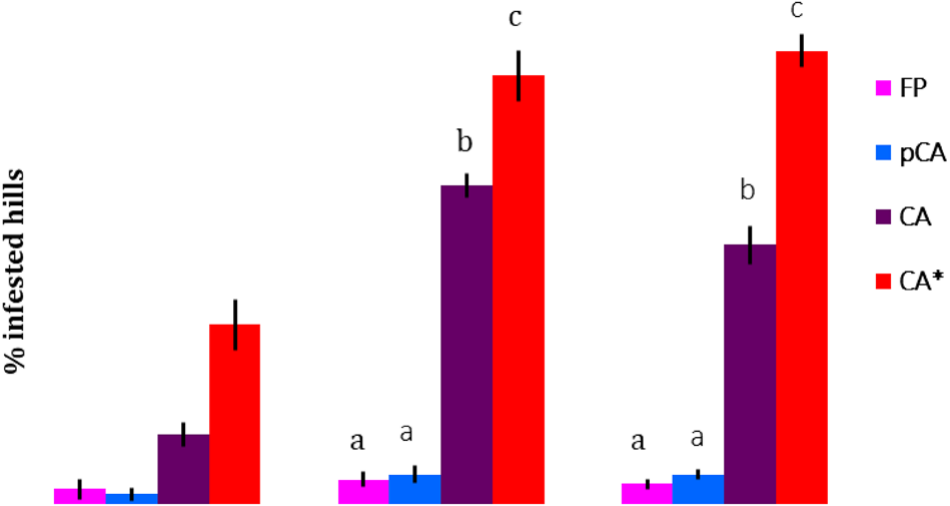
Effect of different tillage cum crop establishment and residue management production systems on rice mealybug, *Brevennia rehi* infestation on paddy tillers from 2015 to 2017. Bars with different letters indicate significant differences among production systems (LSD; *P* < 0.05). * CA-based production system with ZTDSR-Mustard (ZT)-spring maize (ZT) sequences.

A significant alteration in infestation of mealybug on rice was observed in full CA system compared with pCA and FP in the first set of long-term experiments (Fig. 5). Infestation of mealybug in full CA-based system was significantly (*p* < 0.05) higher after the first incidence during 2015 and progressively increased over time (Fig. 5). The highest number of infested hills was observed in both full CA-based systems (52 to 91% infested hills in CA rice–wheat and CA rice-mustard systems, respectively) during 2017. In contrast, a very low incidence (4–6% infested hills) was observed in FP and pCA.

**Figure 6:**
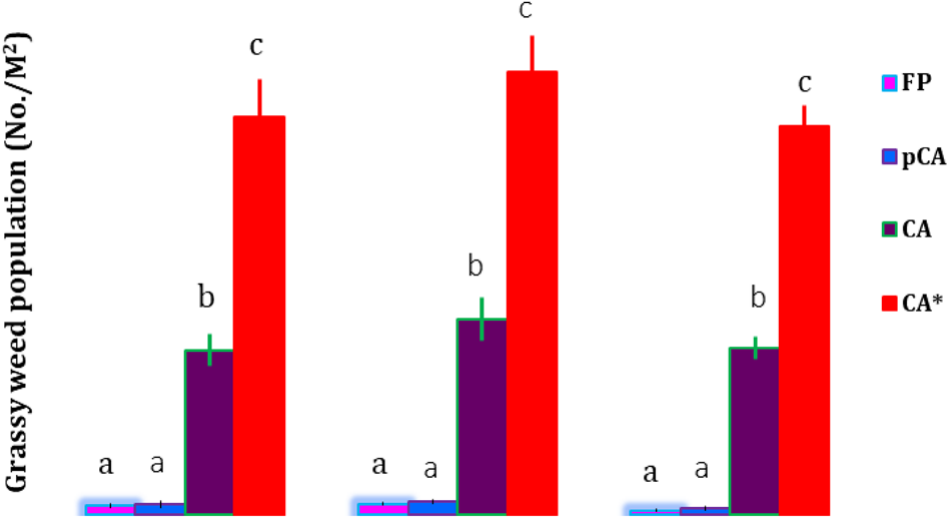
Grassy weed population in fields of different agricultural production systems. Bars with different letters indicate significant differences among production systems (LSD; *P* < 0.05). * CA-based production system with ZTDSR-mustard (ZT)-spring maize (ZT) sequences.

Minimum temperature had played a significant role in population dynamics of rice mealybug from year to year, but the effect was similar among the different CERM practices (Supplementary Table 4). Contrast analysis revealed that tillage (till vs. no-till) and residue management (residue retention vs. non-residue) had no significant effect on mealybug infestation except in 2016 when tillage had a significant negative impact (Table 2). However, grassy weeds were higher in CA systems than in FP and pCA production systems (Fig. 6). Grassy weeds, viz., *Echinochloa colona*, *Echinochloa crus-galli*, *Cynodon dactylon*, *Leptochloa chinensis* and *Panicum repense,* were observed to be comparatively higher in CA. Among CA-based production systems, infestation of mealybug and weeds was two times higher in ZTDSR-mustard (ZT)-spring maize (ZT) system than in the ZTDSR-wheat (ZT)-mungbean (ZT) system.

### Bandicoota rat, *Bandicota bengalensis*

**Figure 7:**
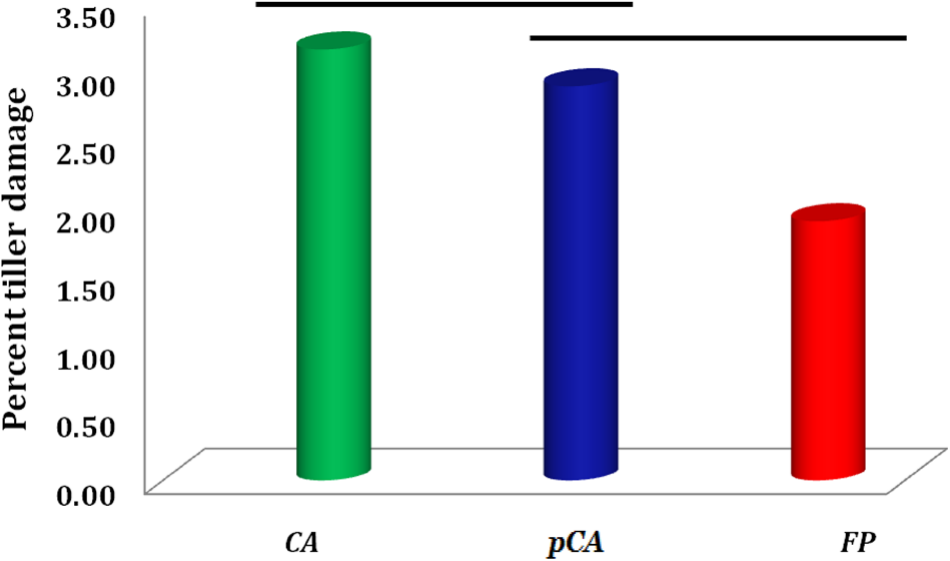
*Percent* tiller damage caused by Bandicoot rats, *Bandicota bengalensis* under diverse tillage and production system. Arrow distance indicates significant differences among production systems (LSD; *P* < 0.05).

The number of live burrows by bandicoot rats was significantly influenced by the different crop establishment-cum-residue management (CERM) and winter crops under rice-fallow system. It was significantly higher in CA plots than in pCA and FP plots (Tables 3, 4). Data on burrow counts indicated a significantly higher number in residue retained plots (4.2 and 13.7 burrows per 62.5 m^2^ during 2019–2020 and 2020–2021, respectively) compared to non-residue plots (1.5 and 7.5 burrows per 62.5 m^2^ in 2019–2020 and 2020–2021, respectively) in CA experiment. The maximum number of burrows was observed in rice-mustard system (15.1 burrows/62.5 m^2^ during 2020–2021), which was the highest in CA plots, followed by pCA plots. Data on damaged tillers in rice by bandicoot rats also indicated a significantly higher infestation in CA (3.4%) and pCA (2.8%) plots as compared to FP (Fig. 7). Overall, the results of bandicoot rats in rice fields indicated a progressively higher infestation in CA-based production systems particularly in rice-mustard-based sequences, and the same was also confirmed by the number of damaged tillers.

**Table 3:** Burrows of bandicoot rats, *B. bengalensis* as influenced by different crop establishment-cum-residue management (CERM) and winter crops in rice-fallow system of eastern India (after 4th years of experimentation: 2019–2020).

CERM	Rodent burrow (no./62.5 m^2^)					Mean
	R–C	R–L	R–SF	R–Li	R–M	
Conservation agriculture (CA)						
R−	2.00 ± 0.58	1.33 ± 0.33	0.33 ± 0.00	1.67 ± 0.33	2 ± 0	1.47^C^
R+	5.33 ± 0.33	8.00 ± 0.58	2.33 ± 0.33	1.67 ± 0.33	3.67 ± 0.33	4.20^A^
Partial conservation agriculture						
R−	2.67 ± 0.33	0.67 ± 0.19	0.67 ± 0.19	1.00 ± 0.00	2.00 ± 0.58	1.40^C^
R+	4.00 ± 0.58	2.67 ± 0.33	1.67 ± 0.67	1.67 ± 0.33	3.00 ± 0.58	2.60^B^
Farmer practices (FP)						
R	2.00 ± 0.58	2.33 ± 0.33	1.33 ± 0.33	2.33 ± 0.33	0.33 ± 0.00	1.67^C^
R+	2.00 ± 0.00	3.00 ± 0.58	2.00 ± 0.58	3.67 ± 0.33	2.00 ± 0.58	2.53^B^
Mean	3.00^A^	3.00^A^	1.39^C^	2.00^B^	2.17^B^	
LSD (*p* = 0.05)	CERM		WC		CERM*WC	
	0.51		0.47		1.15	

R^+^: residue retention (30% RT), R^−^: control; R–C: Rice–Chickpea; R–L: Rice–Lentil; R–SF: Rice–Safflower; R–Li: Rice–Linseed; R–M: Rice–Mustard; Different capital letters (vertical) represent significant variations in CERM; Different (horizontal) capital letters indicate significant variations in different cropping sequences; Values with ± represent standard error of mean.

**Table 4:** Burrows of bandicoot rats, *B. bengalensis* as influenced by different crop establishment-cum-residue management (CERM) and winter crops in rice-fallow system of eastern India (after 5th years of experimentation: 2020–2021).

CERM	Rodent burrow (no./62.5 m^2^)					Mean
	R–C	R–L	R–SF	R–Li	R–M	
Conservation agriculture (CA)						
R−	3.67 ± 0.33	6.00 ± 0.58	0.33 ± 0.00	5.00 ± 0.58	22.67 ± 0.67	7.53^C^
R+	12.67 ± 0.33	11.33 ± 0.33	5.00 ± 0.58	6.67 ± 0.33	32.67 ± 0.67	13.67^A^
Partial conservation agriculture						
R−	4.67 ± 0.33	4.67 ± 0.33	2.67 ± 0.33	4.00 ± 0.58	7.67 ± 0.33	4.73^D^
R+	6.00 ± 0.58	8.67 ± 0.33	5.33 ± 0.33	14.67 ± 0.33	10.00 ± 0.58	8.93^B^
Farmer practices (FP)						
R−	2.00 ± 0.00	0.67 ± 0.00	1.00 ± 0.00	7.33 ± 0.67	7.00 ± 0.58	3.60^E^
R+	3.33 ± 0.33	4 ± 0.58	2.33 ± 0.33	14.33 ± 0.88	10.67 ± 0.33	6.93^C^
Mean	5.39^C^	5.89^C^	2.78^D^	8.67^B^	15.11^A^	
LSD (*p* = 0.05)	CERM		WC		CERM*WC	
	0.62		0.54		1.33	

R^+^: residue retention (30% RT), R^−^: control; R–C: Rice–Chickpea; R–L: Rice–Lentil; R–SF: Rice–Safflower; R–Li: Rice–Linseed; R–M: Rice–Mustard; Different capital letters (vertical) represent significant variations in CERM; Different (horizontal) capital letters indicate significant variations in different cropping sequences; Values with ± represent standard error of mean.

## Discussion

Different pests and their possible reasons for outbursts in long-term CA based production systems in the MIGP of South Asia have been investigated and documented in the present study. CA based techniques are being developed and popularized to address the problems of increasing water scarcity, deteriorating the soil health, declining productivity and profitability, and climate change and for sustainable intensification of rice–wheat cropping system in MIGP and India^11,21,23,24,27^. Our results revealed significant differences in pest populations in different crops after 4–5 years of CA adoption.

A significantly higher number of larvae and pupae of armyworm *M. separata* in wheat was observed in complete CA followed by pCA production system. M. *separata* is a serious cereal polyphagous pest in Asia and Australia^35^. The gregarious feeding of larvae on tender parts causes yield loss or complete crop loss if the pest population increases substantially. Larvae feed on tender foliage and thereby restrict the development of almost all agricultural plants^36^. Damage is caused by early larval instars on young leaves, while mature larvae usually climb up ear head stalks at night. During daytime, larvae rest inside cracks of soil or crop canopy or residue leftover on the soil surface^37^. Tillage had significant negative effects, while residue had significant positive effects on larval population build-up. Larvae and pupae count (2019–2020) indicated a non-significant difference between tillage condition, although it was numerically higher in no-tilled fields than in tilled fields. Pupal number was the highest in the CA-based system and decreased while residue was removed from the soil surface after wheat harvest. Residue retention had a significant positive impact on pupal counts after harvesting wheat. Heavy rainfall followed by drought, flooding and trash mulching has been reported to be population outbreaking factors for *M. separata*^38^. In earlier literature, armyworm (*Pseudaletia unipuncta*) was also reported to be the most damaging insect pest of corn seedlings under reduced tillage (RT) compared to conventionally tilled plots^39^. In no-till conditions, 15% of corn seedlings were infested, whereas, in conventional fields, it was only 1%. They also considered residue retention in RT as a responsible factor and created a favourable environment for the oviposition and survival of larval populations.

In this study, a significantly greater number of larvae and pupae of *M. separata* were observed in residue-retained CA and pCA plots, followed by farmer practices. This can be attributed to the omission of tillage, which otherwise alters the pest population either by physically damaging or exposing them to other predators, such as birds. The retained residue further flourished the pest population by acting as a hiding place for larvae and pupae of *M. separata.* Therefore, continuous no-tillage and residue retention proliferated *M. separata* population in CA-based system and are in good agreement with previous studies^38,39^.

Similar increasing trends for mealybug were also observed in rice, where the maximum damage due to mealybug was reported in CA followed by pCA and very less in FP. Mealybug are considered an occasional serious pest of rice in many Asian countries, America and Australia^40^. In India, it has been considered as a sporadic pest of rice with its restricted distribution in upland and rainfed environments^41^. Mealybug suck plant sap causes leaf curling and ultimately wilting of crop plants^42^. Nymph and adults suck plant sap from the base of plants. Presence of the grassy weeds, i.e., *E.* *colona*, *Echinochloa crusgalli*, *C. dactylon*, *L. chinensis*, *P. repense*, *Paspalum scorbiculatum*, *Eleusine* spp. in rice fields favour population build-up of mealybug by acting as an alternate host and shelter mealybug to survive and multiply in off-seasons^43,44^. Tillage condition and residue retention in different experimental treatments had no direct significant influence on the mealybug in the present study. Indirectly, grassy weeds were higher in number in the CA-based production systems than in the FP and pCA systems. Many authors have reported similar findings and concluded that a higher infestation of grassy weeds is favoured by no or minimum disturbance of soil in CA^26,45,46^. Two times higher infestation of mealybug in maize inclusive cropping sequence was observed compared to mungbean in spring. This might be due to the presence of spring maize stubbles, as maize has also been reported as a preferred host of mealybug^47^.

A significant influence of different CERM and winter crops under rice-fallow production systems was observed on bandicoota rat infestation in rice fields. Live burrows and damaged tillers indicated the presence of bandicoota rats in rice fields, which are a major small mammalian pest of rice and inflict substantial losses^48,49^. Undisturbed (no-tilled) and waterlogged areas with grassy habitats showed a high natural preference for rodent infestation in the field^50^. Increased intensity of rodents in rice-based cropping systems is associated with masting events, changes in abiotic conditions and changes in cropping patterns^51^. Many anthropogenic responses, such as increased intensity of cropping systems with the inclusion of preferred hosts (mustard in the present study), also favour rodent population build-up^49^. The increased intensity of rodents in CA-based production systems may be corroborated by the presence of crop residues in the field and weeds, especially *Digitaria* sp., *Ipomoea aquatica* and *Echinochloa colona,* which have been identified as dominant food items for rodents during off-season months when rice crops are absent^52^.

Our study indicated that CA-based management i.e., retention of crop residue on the soil surface, reduced tillage and in-appropriate cropping sequences could be favourable for the pest population build-up, and the pest status could be a potential threat in future under CA-based systems in MIGP. Study results also revealed tillage-, residue- and weed shift-based pest population outbreaks after 4–5 years of the experimentation. The shift of pest population and associated crop loss due to increase of pest population in the full or partial CA production systems reported in several studies of Indo-Gangetic Plain^13,28^. Thus, the management strategies, i.e., crop rotation, crop residue management through tillage after certain years, weed management in crops and vicinity may be included for complete success of CA production system^26^. The leguminous crops in rice–wheat cropping system could be the potential crops to break the continuous chain of the preferred host plants. Reports suggest that crop diversification with leguminous crops have shown potential for lowering the population load of pests along with improving the soil health and water economy^53^. Overall, CA system has a large number of benefits, including addressing the issues related to land degradation^18^, resource conservation^27^, increasing energy use efficiency and reducing C-footprints and cost of cultivation^27^ in the current context of climate change, but future research is needed for refining this technology in the context of pest status in MIGP.

The yield losses due to the pest outbreak are the most important indicator for determining vulnerability of the system for a particular pest. In our experiments, yield loss due to pest emergence was not consistent (data not presented). The first set of CA experiment which is ongoing for over ten years observed a significant yield loss due to rice mealybug under both CA and pCA but the magnitude of yield reduction maximum for pCA as reported by Mishra et al.^26^. In contrast, in the second set of experiments, incidence of armyworm didn’t have any significant effect on wheat crop yield^21^. Timely management might have prevented any significant crop loss. However, these pests have the potential to create havoc in CA system and could inflict significant yield loss in the near future if proper management strategies are not devised. Therefore, future research should also focus on these emerging issues for the successful adoption of CA by small and marginal farmers of the region.

## Conclusion

Under the changing climatic scenarios, CA-based management interventions are required for rice-based cropping systems and should be promoted to overcome the adversities of climate change on crop productivity. Long-term crop management activities such as tillage, crop residue and cropping sequences have great influences on pest incidence and activity. Potential threats of pests and their possible reasons for long-term CA in MIGP were investigated in the present study. Armyworm, *M. separata* in wheat and mealybug, *B. rehi* and Bandicoota rats, *B. bengalensis* in rice were reported at an epidemic level under different long-term CA systems. These reported pests could pose the major challenges to crop production under environmentally benign CA in near future. No-tillage with residue cover on soil surface has a harbouring and positive impact on populations of these pests. Tillage and residue were also found to bring changes in surrounding habitats, which were favourable for pest population outbreaks. The occurrences of these pests indicate that to bring full benefits of CA, pest management strategies need to be revised with respect to tillage, residue retained on soil surface, grassy weeds in the fields and cropping sequences. In addition, future studies in long-term experiments of CA should focus on changes in major pests and their natural enemies across the years to obtain more in-depth clarity on pest population behaviour and dynamics.

## Supplementary Information

Supplementary Information.
