# Supplementary material for: Outburst of pest populations in rice-based cropping systems under conservation agricultural practices in the middle Indo-Gangetic Plains of South Asia
Source: Sci Rep. 2022 Mar 8;12:3753. doi: 10.1038/s41598-022-07760-w (PMC8904590; doi:10.1038/s41598-022-07760-w)
Supplement: Supplementary file 1 — Supplementary Information. [file 41598_2022_7760_MOESM1_ESM.doc]

**Outburst of pest populations in rice-based cropping systems under conservation agricultural practices in the middle Indo-Gangetic Plains of South Asia**

Rakesh Kumar^a*^, Jaipal Singh Choudhary^b*^, Janki Sharan Mishra^c*^, Surajit Mondal^a^, Shishpal Poonia^d^, Mohammad Monobrullah^a^, Hansraj Hans^a^, Mausam Verma^a^, Ujjwal Kumar^a^, Bhagwati Prasad Bhatt^e^, Ram Kanwar Malik^d^, Virender Kumar^f^, Andrew McDonald^g^

^a^ICAR-Research Complex for Eastern Region, Patna, Bihar, India

^b^ICAR-Research Complex for Eastern Region, FSRCHPR, Ranchi, Jharkhand, India

^c^ICAR-Directorate of Weed Research, Jabalpur, Madhya Pradesh, India

^d^Cereal Systems Initiative for South Asia (CSISA)-CIMMYT, Patna, India

^e^Indian Council of Agricultural Research, New Delhi, India

^f^International Rice Research Institute, Los Banos, Philippines

^g^Soil and Crop Sci. Sec., School of Integrative Plant Sci., Cornell University, Ithaca, NY, USA

*Corresponding authors: [choudhary.jaipal@gmail.com](mailto:choudhary.jaipal@gmail.com) (Jaipal Singh Choudhary)

[rakeshbhu08@gmail.com](mailto:rakeshbhu08@gmail.com) (Rakesh Kumar)

[jsmishra31@gmail.com](mailto:jsmishra31@gmail.com) (Janki Sharan Mishra)

**Supplementary Table 1**. Details of tillage, seeding/planting methods, crop rotation and residue management under different treatments. (First set of CA experiments)

| Treatment Name |  | Traditional agriculture | Partial conservation agriculture 1 | Full conservation agriculture | Partial conservation agriculture 2 |
| --- | --- | --- | --- | --- | --- |
| Treatment abbreviation |  | TA | pCA1 | fCA | pCA2 |
| Drivers of change |  | Business as usual (farmers' practice) (FP) | To increase production and income through best  management practices | To deal with the rising scarcity of water, energy and labour, degrading soil health (CA practice) | Futuristic, intensified and diversified for food and nutritional security and farm profitability |
| Tillage method | 2009–2014 | Wheat – CT  Rice – conventional tillage (CT) followed by puddling | Wheat – no–tillage (ZT)  Mung bean – ZT  Rice – CT (puddled) | Wheat – ZT  Cowpea – ZT  Rice – ZT | Potato/Maize – CT  Cowpea – ZT  Rice – CT (unpuddled) |
|  | 2014–2019 |  | Wheat – ZT  Mung bean – ZT  Rice – CT followed by machine transplanting (unpuddled) | Wheat – ZT  Mung bean – ZT  Rice – ZT | Mustard – ZT  Maize – ZT  Rice – ZT |
| Seeding/ planting method | 2009–2014 | Wheat – drill seeding  Rice – transplanting | Wheat – drill seeding  Mung bean – drill seeding  Rice – transplanting | Wheat – drill seeding  Cowpea – drill seeding  Rice – drill seeding (DSR) | Potato/maize – dibbling  Cowpea – drill seeding  Rice – transplanting |
|  | 2014–2019 |  | Wheat – drill seeding  Mung bean – drill seeding  Rice – transplanting | Wheat – drill seeding  Mung bean – drill seeding  Rice – drill seeding (DSR) | Mustard – drill seeding  Maize – drill seeding  Rice – drill seeding (DSR) |
| Crop rotation | 2009–2014 | Wheat – fallow – rice | Wheat – Mung bean – rice | Wheat – cowpea – rice | Potato+maize – cowpea – rice |
|  | 2014–2019 |  | Wheat – Mung bean – rice | Wheat – Mung bean – rice | Mustard – maize – rice |
| Crop residue management |  | Rice/Wheat – removed from ground level | Wheat – removed  Rice – removed  Mung bean – retained full and incorporated | Wheat – 1/3^rd^ retained  Rice – 1/3^rd^ retained  Cowpea – retained full | Potato – full, incorporated  Maize – 1/3^rd^ retained  Cowpea – full, incorporated  Rice – 1/3^rd^ incorporated |
|  | 2014–2019 |  |  | Wheat – 1/3^rd^ retained  Rice – 1/3^rd^ retained  Mung bean – retained full | Mustard – 1/3^rd^ retained  Maize – 1/3^rd^ retained  Rice – 1/3^rd^ retained |

**Supplementary Table 2.** Establishment of different tillage cum crop establishment and residues management practices in rice-wheat-mung bean cropping system under middle Indo-Gangetic plains (MIGP) of India. (Second set of CA experiments)

| Parameter | **Production systems** | **Farmer practices (FP)** | | | **Partial conservation agriculture**  **(pCA)** | | | **Conservation agriculture (CA)** | |
| --- | --- | --- | --- | --- | --- | --- | --- | --- | --- |
|  | Crop | RPTR-BCW - ZTG | LPTR-CTW-ZTG | | CTMTR-ZTW-ZTG | CTDSR-ZTW-ZTG | SRI-SWI-ZTG | ZTMTR-ZTW-ZTG | ZTDSR-ZTW-ZTG |
|  |  | Sc1 | Sc2 | | Sc3 | Sc6 | Sc5 | Sc4 | Sc7 |
| Tillage | Rice | Cultivator: 2 passes (dry tillage: DT)  Rotavator: 1 pass (wet tillage: WT) | | | Cultivator: 2 passes  Rotavator: 1 pass | Cultivator: 2 passes (DT), Rotavator: 1 pass (WT) | | Zero till (flooding before-transplanting) | Zero-till |
|  | Wheat | Cultivator: 2 passes  Rotavator: 1 pass | | | Zero till | | Cultivator: 2 passes  Rotavator: 1 pass | Zero till | |
|  | Mung bean | Zero till | | | | | | | |
| Crop establi-shment | Rice: |  | |  |  |  |  |  |  |
|  | Transplanting/ Seeding | Manual | | Manual | Machine | Drill seeding | Manual | Machine | Drill seeding |
|  | Seedling age | 25 days | | 25 days | 18 days | - | 12 days | 18 days | - |
|  | Spacing (cm) | Random | | 20 x 15 | 23 x 14 | 22.5 cm row spacing | 25 x 25 | 23 x 14 | 22.5 cm row spacing |
|  | Wheat | Broadcasting | | Drill seeding | Drill seeding | Drill seeding | Manual | Drill seeding | Drill seeding |
|  | Mung bean | Drill seeding with Happy Seeder | | | | | | | |
| Residue  manage-ment | Rice | ~30% mixed | | | ~30% retained | | ~30% mixed | ~30% retained | |
|  | Wheat | ~30% residues retained | | | | | | | |
|  | Mung bean | 100% residues incorporated | | | | | | | |

**Supplementary Table 3.** Treatment description and abbreviations used in the study. (Third set of CA experiments)

| **Treatment/**  **abbreviation** | **Description** |
| --- | --- |
| CA | Rice was directly sown (22.5-cm row spacing) in the main field under zero-tillage condition by Happy Seeder (zero–tillage seed cum fertilizer drill). A uniform seed rate of 30 kg ha^-1^ used in ZTDSR treatment (~ 90 plants m^-2^). Pre-established weeds were controlled through pre-sowing application of glyphosate (41% EC). |
| CA | Sowing and weed management were same as ZTDSR. Rice was manually harvested and 20-cm rice stubbles were retained as a part of the treatment. |
| pCA | Twenty-one day old seedlings were manually transplanted in unploughed main field with a spacing of 20 cm × 15 cm. One day before transplanting field was irrigated (~10 ha-cm) to make the soil soften and loose. Pre-established weeds were controlled through pre-sowing application of glyphosate (41% EC). |
| pCA | Same as ZTTPR with 20-cm rice residues retention (~30%). |
| FP | Field was prepared by two passes of dry-harrowing followed by planking. Wet-tillage was done by tractor-drawn rotavator under standing water (~10 cm). Manual transplanting of twenty-one day old rice seedling was done with a spacing of 20 cm × 15 cm. |
| FP | Same as PTR with 20-cm rice residue retention. |

**Supplementary Table 4.** Relationship between mean monthly weather parameters and insect pest population in different crop establishment-cum-residue management (CERM)

| CERM | *Mythimna separata* | | | | | | Rice mealybug, *Brevennia rehi* | | |
| --- | --- | --- | --- | --- | --- | --- | --- | --- | --- |
|  | Larva | | | Pupa | | |  |  |  |
|  | Tmax | Tmin | Rainfall | Tmax | Tmin | Rainfall | Tmax | Tmin | Rainfall |
| Conservation agriculture (CA) | 0.80 | **0.96** | 0.14 | **0.98** | **-0.98** | -0.31 | -0.33 | **-0.99** | 0.16 |
| Partial conservation agriculture (pCA) | 0.80 | **0.96** | 0.14 | **0.99** | **-0.97** | -0.32 | -0.45 | **-0.99** | 0.60 |
| Farmer practices (FP) | 0.83 | **0.97** | 0.09 | **0.97** | **-0.98** | -0.41 | 0.65 | **-0.98** | -0.55 |

*Values shown in bold are significant at 0.05 levels.
